# Editorial: CRISPR advancement in cancer research and future perspectives

**DOI:** 10.3389/fonc.2023.1173527

**Published:** 2023-03-09

**Authors:** Alessio Biagioni, Reza Mohammadinejad

**Affiliations:** ^1^ Department of Experimental and Clinical Biomedical Sciences “Mario Serio”, University of Florence, Firenze, Italy; ^2^ Research Center of Tropical and Infectious Diseases, Kerman University of Medical Sciences, Kerman, Iran

**Keywords:** cancer therapeutics, gene editing, gene therapy, gene delivery, CRISPR

CRISPR (clustered regularly interspaced short palindromic repeats) is a prokaryotic adaptable immune system used by many bacteria and archaea to avoid foreign nucleic acids genome integration (1). Its high usability and relatively low cost are two features that have led to CRISPR gaining huge success in gene editing, overcoming previous technologies such as TALEN, meganucleases, and ZFNs (2). According to the basic research, CRISPR and, in particular, the relative CRISPR-associated proteins (Cas) have slowly evolved to be applicable to clinical practice, leading the worldwide scientific community into the challenge of using such a technology as a therapeutic treatment in several genetically derived pathologies (3). In recent years, CRISPR-Cas systems have been widely used in cancer research. This unique gene-editing technique is applied to correct gene mutations in cancer cells, discover therapeutics, reveal gene functions, engineer chromosome aberrations, modulate non-coding regions/non-coding RNAs, interrogate chromatin regulation, and build cancer models (4, 5). Identifying new druggable genetic alterations to overcome therapeutic resistance is another emerging benefit of CRISPR-Cas technology. In this regard, Hou et al. reviewed the recent advances in utilizing CRISPR-Cas screening and patient-derived models to understand the tumor resistance mechanisms, novel exploitable targets, and potential strategies to improve current modalities in molecular targeted therapies. They highlighted the role of Capicua, neurofibromin 1, tankyrase, and RIC8 guanine nucleotide exchange factor A in decreasing the efficacy of EGFR inhibitors. Hou et al. also focused on the mechanisms of resistance to anti-angiogenic agents, immune checkpoint inhibitors, immune cell therapies, and cancer vaccines. Conducting research on these signaling pathways shaded light on precision oncotherapy, next-generation anti-cancer therapeutics have been discovered, and new combination therapeutic strategies developed (6). Moreover, Zhang et al. investigated the tumor resistance mechanisms to poly(ADP-ribose) (PAR) polymerase inhibitors (PARPi). They performed a genome-wide CRISPR screen to evaluate their hypothesis related to these FDA-approved anti-cancer drugs. Using this strategy, TBL1XR1, which stabilizes SMC3 on chromatin and promotes γH2AX spreading along the chromatin, has been found to regulate the sensitivity of prostate cancer lines to PARPi. Interestingly, TBL1XR1-SMC3 double-knockdown cells had comparable PARPi sensitivity to TBL1XR1 or SMC3 knockdown cells. Furthermore, these double-knockdown cells had more sensitivity to PARPi than WT cells. Amongst the most recent CRISPR designs, we report a study performed by Biagioni et al. on the Cas9-mediated knockout of uPAR, which is a globular protein tethered to the external surface of the cell membrane, involved in several typical cancer features such as survival, invasion, migration, angiogenesis, and intra-tumor recruitment of inflammatory cells, They evidenced in melanoma and colon carcinoma cell lines a significant impairment of cancer growth, both *in vitro* and *in vivo*, and the unexpected acquisition of stem-like markers that might be compatible with the “molecular sponge” function of uPAR 3’UTR mRNA. Indeed, uPAR 3’ UTR mRNA was demonstrated to be capable of attracting some miRNAs, including miR146a, thus blocking their action when the uPAR transcript is strongly expressed, a phenomenon that was thought to be responsible for uPAR-dependent EGFR inhibition. These gene-edited cell lines also underwent a significant metabolic rewiring, during which was observed an increased number of mitochondria in the two melanoma cell lines and an immature biogenesis of mitochondria in the colon carcinoma one. Such dysregulation in the respiratory apparatus led to a significant increase in the mitochondrial spare respiratory capacity paired with the upregulation of GLS2 and decreased glycolysis (7). A broader use of such a powerful method was formed by Peng et al. by developing four hypermutator or ultramutator phenotype cell lines through the introduction of several variants of the MutS Homolog 2 (MSH2) gene and DNA polymerase epsilon (POLE) gene *via* CRISPR/Cas9 technology. In that way, they were able to generate a novel set of formalin-fixed and paraffin-embedded samples with different tumor mutational burden values as reference materials for the validation, verification, internal quality control, and proficiency testing of the mutations assessment. Out of the basic research field, CRISPR has already been widely used in oncological therapy mainly by the generation of chimeric antigen receptor T cells (CAR-T). Indeed, engineering T-cell receptors, and making them capable of identifying and destroying cancer cells to avoid healthy tissues, has opened a new frontier for immunotherapy, giving clinicians a new powerful tool to face hard-to-treat neoplasias such as lung cancer (8), leukemia, lymphoma, and myeloma (9). In conclusion, CRISPR is proving to be increasingly essential in all research fields to better understand the molecular mechanisms of tumor progression and metastasization and to find new therapeutic options that are exploitable for cancer histotypes that currently lack proper treatments.

## Author contributions

All authors listed have made a substantial, direct, and intellectual contribution to the work and approved it for publication.
